# The Spine: A Strong, Stable, and Flexible Structure with Biomimetics Potential

**DOI:** 10.3390/biomimetics4030060

**Published:** 2019-08-30

**Authors:** Fabio Galbusera, Tito Bassani

**Affiliations:** Laboratory of Biological Structures Mechanics, IRCCS Istituto Ortopedico Galeazzi, 20161 Milan, Italy

**Keywords:** spine, vertebrates, lordosis, energy consumption, flexibility, stability

## Abstract

From its first appearance in early vertebrates, the spine evolved the function of protecting the spinal cord, avoiding excessive straining during body motion. Its stiffness and strength provided the basis for the development of the axial skeleton as the mechanical support of later animals, especially those which moved to the terrestrial environment where gravity loads are not alleviated by the buoyant force of water. In tetrapods, the functions of the spine can be summarized as follows: protecting the spinal cord; supporting the weight of the body, transmitting it to the ground through the limbs; allowing the motion of the trunk, through to its flexibility; providing robust origins and insertions to the muscles of trunk and limbs. This narrative review provides a brief perspective on the development of the spine in vertebrates, first from an evolutionary, and then from an embryological point of view. The paper describes functions and the shape of the spine throughout the whole evolution of vertebrates and vertebrate embryos, from primordial jawless fish to extant animals such as birds and humans, highlighting its fundamental features such as strength, stability, and flexibility, which gives it huge potential as a basis for bio-inspired technologies.

## 1. Introduction

The spine is the defining feature of vertebrates, a large group of animals with more than 66,000 extant species [1]. The spine has the main function of protecting the spinal cord from traumas and excessive straining which may occur during body motion [2]; in addition, the stiffness and stability of the spine provide the capability of supporting the body weight and the forces generated by muscles to allow for locomotion and physiological activities, especially in animals living in the terrestrial environment where the gravitational load is not partially supported by buoyancy.

Vertebrates colonized the whole world as they thrive in many environments, such as seas, rivers, swamps, forests, grassland, mountains, and deserts [3]. Terrestrial vertebrates employ a number of locomotor behaviors, such as quadrupedal and bipedal walking and running, hopping, and brachiation, as well as flight, thus demonstrating the biomechanical validity and efficiency of the body plan based on a vertebral column [3].

This narrative review describes the fundamental anatomy and features of the spine, highlighting its strength, stability, and flexibility, first from an evolutionary and then from an embryological perspective. Finally, a brief overview about the potential of the spine as a basis for bio-inspired technologies, especially in the field of structural engineering, is reported.

### Vertebral Anatomy

**Figure 1:**
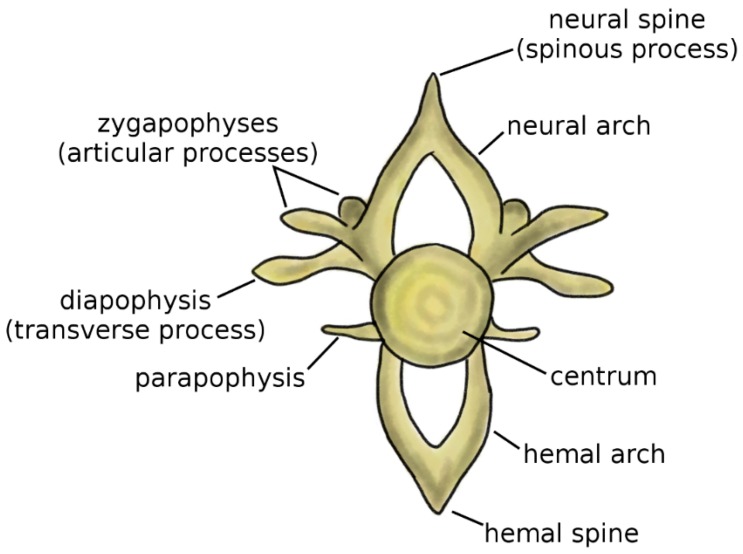
Schematic representation of a vertebra. Adapted from Reference [6].

Vertebrae are the fundamental and characterizing components of the spine. Although the anatomy of the vertebra widely varies among vertebrate species, some recurring elements can be identified (Figure 1) [4,5]. The centrum, or vertebral body, represents the structural core of the vertebra. Two arches, the neural arch in the dorsal direction and the hemal arch in the ventral direction, project from to the centrum; the spinal cord occupies the space between the posterior wall of the centrum and the inner surface of the neural arch. The tips of the two arches are named the neural spine and hemal spine, respectively; in the clinical literature, the neural spine is most commonly referred to as spinous process. Not all vertebrates possess the hemal arch and spine; even in species which have them, they may not be present in all spine regions. The zygapophyses, or articular processes, protrude from the neural arch caudally and cranially. The diapophyses, commonly named transverse process in the clinical literature, project laterally from the neural arch, whereas the parapophyses project from the centrum [5].

## 2. A Biomechanical Perspective on Spine Evolution

### 2.1. Jawless Fish

The presence of a spine is shared by all extant vertebrates, including the oldest surviving class of *Myxini* (hagfish), which are jawless fish believed until recently to possess a skull but not a vertebral column [5,7,8]. Although no signs of vertebrae can be found during a macroscopical anatomical examination of hagfish, a recent genetic study determined that a rudimentary spine, which develops in the embryo with the same mechanisms observed in the other vertebrates, exists [8].

The living jawless fish, i.e., hagfish and lampreys (cyclostomes), were long believed to be “degenerated” forms of ostracoderms, the armored jawless fish which were predominant in the Ordovician and Silurian (485 to 419 million years ago), after losing the dermal skeleton and paired fins [9,10], as well as the spine in the case of the hagfish. The discovery of the first lamprey fossil, *Mayomizon pieckoensis* [11], which shared many characteristics with the extant species, and was almost contemporary of the late ostracoderms, started to cast doubts on the belief that cyclostomes could have derived through “degeneracy” from ostracoderms [9,12]. Although recent studies confirmed that the ancestors of lampreys and hagfish were more complex than the living species [13], research seems to indicate that cyclostomes split from ostracoderms before the appearance of bone tissue and were, thus, not a “degenerated” form of them [12,14].

Lampreys are currently an important model for the study of the evolution of vertebrates since they retain primitive characteristics from the ancestral vertebrates, but they are at the same time close relatives to animals having a proper bony skeleton such as modern fish, the osteichthyes [15], such as for example *Perca fluviatilis*. Lampreys can be subjected to examination with numerous experimental techniques [16]. They do not possess a bony skeleton but have a cartilaginous cranium, a fused branchial basket to support the pharynx [17], and small rudimentary vertebrae, i.e., neural arches connected to the notochord, a long fibrous structure which follows the cranio-caudal axis of the body [9]. The presence of the notochord in any stage of the life cycle, independently of the presence of a proper spine or not, together with other features (dorsal nervous system, pharyngeal slits), defines the phylum *Chordata*, which includes hagfish, lampreys, vertebrates, and tunicates [18].

In both lamprey and hagfish, vertebral elements have a negligible ability to provide biomechanical support to the body; this function is carried out by the notochord, which remains prominent even in adult individuals [19,20]. Although there is no evidence of vertebral elements in ostracoderm fossils, they also possibly possessed rudimentary vertebral elements [5].

### 2.2. Jawed Fish

The first fish with opposing jaws appeared in the Silurian and became dominant in the Devonian (419–358 million years ago) [5]. Placoderms, which had their head and part of the trunk protected by bone plates, were among the first jawed fish; a recent study suggested that some placoderms may have had a bony jaw skeleton, thus disproving the consolidated theory that modern bony fish evolved from jawed ancestors with a cartilaginous skeleton, the chondrichthyes, today represented by sharks and rays [21].

**Figure 2:**
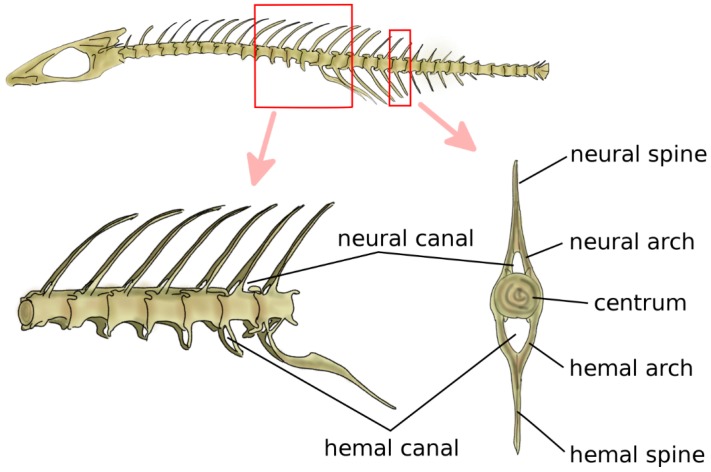
Schematic representation of the spine and vertebral anatomy of a ray-finned fish. Adapted from Reference [32].

Jawed fish are the first animals, from a phylogenetic point of view, which have cartilaginous or bony vertebrae [9]. Vertebrae in jawed fish include the neural arch and spine, the centrum, and the hemal arch and hemal spine in the caudal region, which protect the caudal artery and vein (Figure 2) [5]. The centrum can be either fused or be composed of a variable number of bones, such as intercentra and pleurocentra. Ribs are connected to the vertebrae, most commonly in two sets, i.e., ventral and dorsal. Fish do not possess a sternum. Bone density is generally relatively low; indeed, skeletal loads during locomotion are limited by the buoyant support in the aquatic environment [22,23].

In primitive jawed fish, both with a cartilaginous or bony skeleton, the notochord retains the major role of structural support for the body [24]. Findings of early chondrichthyes such as *Acanthodes sulcatus* showed vertebral elements, namely, neural and hemal arches, and no signs of developed centra [25,26]. Phylogenetically older teleosts, such as sturgeons, e.g., *Acipenser sturio*, and living sarcopterygii, i.e., lobe-finned fish such as *Latimeria chalumnae*, have a prominent notochord connected to bony elements such as ribs, neural and hemal arches, and spines, with no evident centra [27,28]. Fossils of *Saurichthys curionii*, an early ray-finned fish, showed non-ossified vertebral centra and well-defined neural and hemal arches not fused in the midsagittal plane [29]. Modern bony fish such as *Danio rerio* have well-defined centra [30] which provide the main structural support to the skeleton; the notochord is retained but most commonly segmentally interrupted, and acts as a connective pad between the adjacent centra, providing stiffness in bending [31] (Figure 2). Despite their remote split with teleosts and their lack of bone tissue, modern sharks share a similar spinal anatomy with prominent centra with a major structural role; however, the centra are hollow, and the notochord is continuous although constricted at the center of each centrum [5].

### 2.3. Tetrapods

In the Devonian period, aquatic vertebrates started to colonize the terrestrial environment. At first, transitional forms between lobe-finned fish and four-limbed animals, i.e., tetrapods, conducted a predominantly aquatic or semiaquatic life in shallow water and swamps, where the limbs were effective in enhancing the locomotion; outside of water, the stride was slow and likely required a high energy expenditure [33]. The limbs, derived from the paired fins, were connected to the spine by means of the shoulder and the pelvic girdles [34]. Whereas in fish the shoulder girdle (the articulation between the pectoral fins and the rest of the skeleton) is part of the skull complex, shoulders are detached from the skull in tetrapods, resulting in a higher degree of mobility of forelimbs and neck at the expense of a deterioration of the hydrodynamic performance [35,36].

Regarding the hindlimbs, which are homologous to the pelvic fins in fish [37], in early tetrapods, they were connected to the spine through two bony elements which formed a girdle by articulating at the pubic symphysis [5]. A solid connection between the pelvis and the spine able to resist the large propulsive forces generated by the hindlimbs was ensured by the sacrum [37], a set of fused vertebrae and ribs which articulated directly with the pelvic bones. The sacrum first evolved in basal tetrapods which conducted an aquatic life, such as *Acanthostega.* In these tetrapods, the pelvic girdle and the spine were mechanically connected by means a ligamentous complex, probably only allowing for brief periods of land locomotion [38]. Other early tetrapods such as *Ichthyostega* had a more robust sacrum, ilium, and ischium, as well as wider insertions for the muscles acting in terrestrial locomotion, which permitted longer periods of moving between different water sources [36].

**Figure 3:**
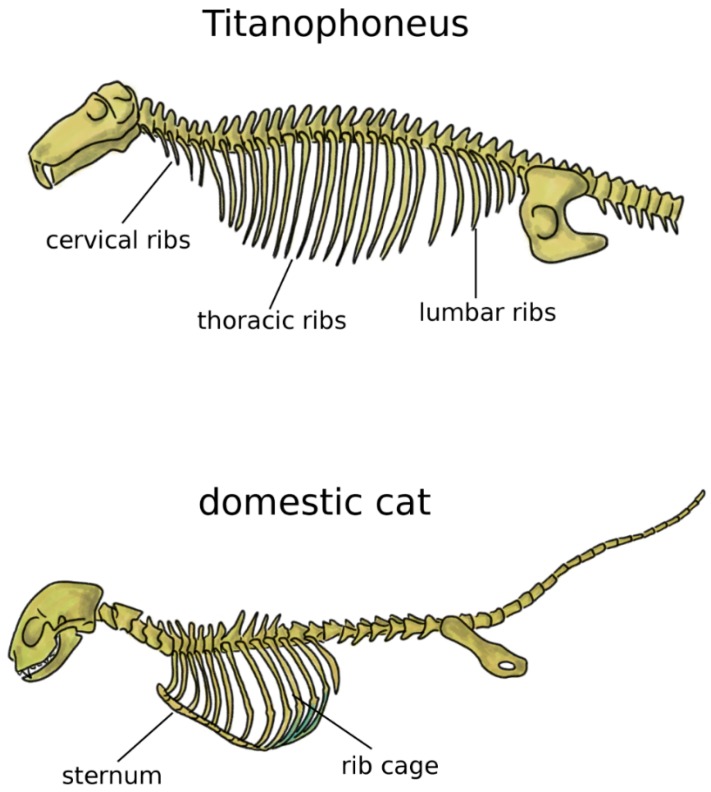
Axial skeleton of a reptile, the extinct *Titanophoneus* (top, tail not completely shown) and a mammal, *Felis catus* (bottom), highlighting the presence of cervical, thoracic, and lumbar ribs in the former, and of the rib cage and sternum in the latter.

In addition to the limbs, terrestrial life and locomotion forced extraordinary changes to the structure of the axial skeleton of tetrapods. Since their bodies could not count on the buoyant support of water anymore, the loads acting on vertebrae and notochord dramatically increased [33]. Whereas in fish and transitional vertebrates vertebral centra were frequently composed by several bones linked by cartilage and connective tissue, tetrapods developed sturdy fused vertebral bodies with higher bone density. In tetrapods, the structural role of the notochord was generally minor, even if this structure showed a large variability among early ones [38,39]. Robust dorsal ribs articulated with both the centrum and the neural arch replaced the thin ribs found in fish [38]; the ventral tips of the ribs were connected in the sternum, which mechanically closed the rib cage and offered stable insertion locations for chest muscles (Figure 3) [5]. Some extant tetrapods such as snakes and turtles do not possess a sternum, whereas it is commonly prominent and even keeled in birds, in order to sustain the high forces generated by the powerful muscles of the wings, as well as to provide them sturdy insertion points [5,40]. Reptiles possess cervical ribs [41], as well as birds, albeit smaller and fused with the vertebrae [42] (Figure 3). In mammals, ribs are present only in the thoracic spine, whereas cervical and lumbar vestigial remnants are fused with the transverse processes [5].

**Figure 4:**
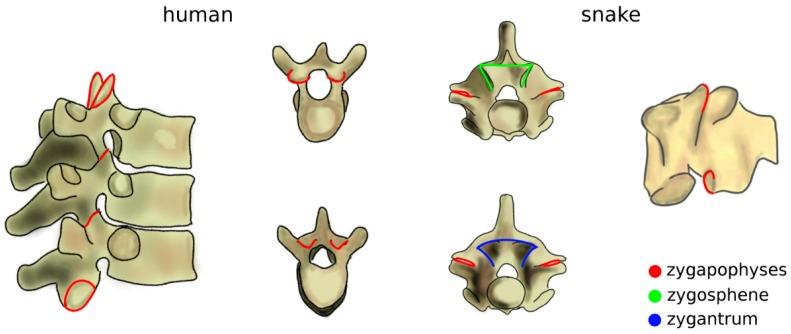
Zygapophyses (in red) in the human thoracic spine (left) and in a snake vertebra (right). In the latter, the zygosphene–zygantrum joints are also highlighted (in green and blue). Top: cranial view; bottom; caudal view. Part of the figure was adapted from Reference [49].

The increase of mechanical loads in the spine due to the transition to terrestrial life also included a rise of the bending moments [43]. Zygapophyses, interlocking processes which limit the flexibility of the spine in extension which already existed in fish, further evolved as a passive mechanism, effective in preventing sag (Figure 4) [44]. The zygapophyses also restrict twisting motion [45], which became critical as terrestrial locomotion also involves high torsional loads which are significantly lower in aquatic life [43]. Snakes and several other reptiles possess an accessory set of articulations with a function similar to that of the zygapophyses, the zygosphene–zygantrum joints, which further stabilize the spine in torsion while not affecting flexion–extension and lateral bending (Figure 4) [46]. Another accessory articulation, the hyposphene–hypantrum, was observed in fossil reptiles and probably had the function of stabilizing the spine in compression, facilitating dinosaur gigantism at the cost of reduced flexibility [47]. Interestingly, in many cetaceans, marine mammals evolved from terrestrial progenitors, whereby anatomical structures with a peculiar non-aquatic function such as zygapophyses and the pelvis were lost, restricted to some spinal regions, or even reduced to vestigial remnants [48].

### 2.4. Spine Regionalization and Specialization

The vertebral column of fish is differentiated in two regions: the trunk vertebrae, which cover the portion of the body between the skull and the tail, and the caudal spine [50]. Trunk vertebrae are connected to the ribs and feature neural arches and spines, whereas caudal ones have no ribs; however, in addition to the neural arch, they possess a hemal arch protecting the caudal artery and vein [5]. In early tetrapods such as *Ichthyostega*, the presence of the shoulders determined the appearance of the neck, i.e., the cervical spine; cervical vertebrae were rather similar to those in the trunk [51]. In contrast to most sarcopterygii, which possess a series of dermal bones connecting the skull to the shoulder girdle, in early tetrapods, the bony connection between head and upper limbs existed only in the cervical spine [52,53]. The sacrum divided the caudal spine from the trunk [53].

**Figure 5:**
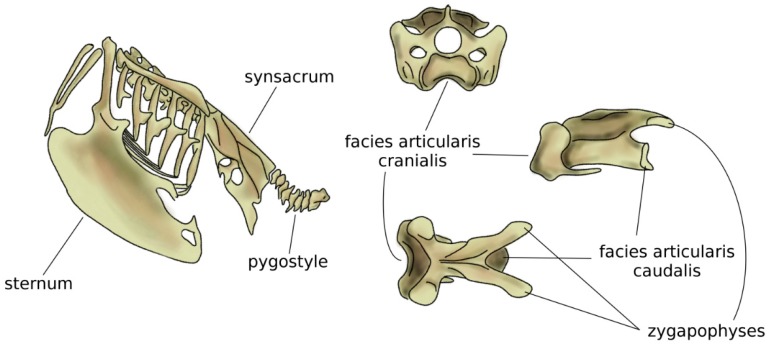
Schematic view of the trunk skeleton in a bird (left). On the right, from top to bottom: cranial, sagittal, and dorsal views of a cervical vertebra of a seagull. The facies articularis cranialis articulates with the facies articularis caudalis of the next vertebra; their shapes allow for a large range of motion. Adapted from Reference [62].

In amniotes, i.e., reptiles, birds, and mammals, spinal regions typically comprise the cervical, thoracic, lumbar, sacral, and caudal spines [54], which show a high specialization aimed at optimizing their function based on the environment and on the behavior of the species. The anatomy of the cervical spine shows a large variability among tetrapods; whereas the number of vertebrae is highly variable in reptiles and birds, all mammals possess seven cervical vertebrae, with the only exception of sloths (which have 5–9 cervical vertebrae depending on the species) and manatees (six cervical vertebrae) [55]. However, in all amniotes, the first two cervical vertebrae, named atlas and axis, developed a peculiar anatomy which allows for large flexibility in torsion, facilitating the head motion [56,57]. The neck of birds is generally highly mobile, due to both a high number of vertebrae (13 to 25) and the shape of the vertebrae themselves [58], in particular the facies articularis cranialis and caudalis which join the adjacent vertebral bodies [59]. The large head mobility effectively compensates for the immobility of the eyes and improves the dexterity of the beak while feeding and performing other tasks (Figure 5). Moreover, the cervical flexibility is fundamental in head-bobbing, i.e., the capability of stabilizing the head during terrestrial locomotion and flight [59].

Compared to the neck, birds have a fused middle and lower spine, in which the sacrum is fused with lumbar vertebrae, the posterior aspect of some thoracic vertebrae, and possibly caudal ones [5,58]. The large but light bony element resulting from the fused vertebrae is named the synsacrum and provides strength and a stable axis to the body during flight (Figure 5) [60]. In several bird species, pelvic bones are also fused to the synsacrum, resulting in a large pelvis complex with an elaborate shape [42]. Caudal vertebrae are fused in the pygostyle, to which the tail feathers are attached, which provides advantages in flight control [61].

Mammals exhibit a strong differentiation between the thoracic and the lumbar spine, with the former being generally stiff and the latter being flexible and adapted to the specific locomotion schemes [63,64]. Although the number of vertebrae in mammals is relatively homogeneous [65], the high morphological variability of vertebrae allows the mammal spine to cover a wide range of biomechanical properties, thus allowing it to adapt to several behaviors such as running, jumping, swimming, and crawling along tree branches [66]. A recent paper demonstrated that, although phylogeny and size constraints are fundamental determinants of the morphology of the lumbar spine in mammals, locomotor ecology factors had a major influence as well [63]. In the paper, the authors described strong differences in species adapted to asymmetric gait, i.e., running, such as *Equus caballus* and *Crocuta crocuta*, which exhibited elongated neural spines and transverse processes, with respect to species with fossorial, scansorial, and arboreal behaviors, like *Talpa europea* and *Ailurus fulgens*, which have more developed articular processes providing stable insertions for muscles used for digging and climbing.

### 2.5. Bipedalism

Although the majority of vertebrates use quadrupedal posture and gait, several bipedal species exist or previously existed. The adoption of bipedalism involved profound changes in the biomechanical environment of the spine due to the altered loading conditions and motion requirements, which resulted in various strategies adopted by different species.

All extant birds are bipeds when not flying, and inherited this characteristic from their dinosaur ancestors [67]. Several lizard species adopt a bipedal gait when high speed is required, for example, for escaping from predators [68]. Kangaroos and several rodents move by hopping in an erect posture [69,70]. Furthermore, some primates which conduct a mostly arboreal life such as gibbons have a bipedal posture when on the ground [71]. Great apes such as chimpanzees and gorillas most commonly walk on four limbs but can adopt a bipedal gait for short routes [72]. Although humans can also move in a quadruped fashion, such as in the infant age, their favored gait and posture are notoriously bipedal.

**Figure 6:**
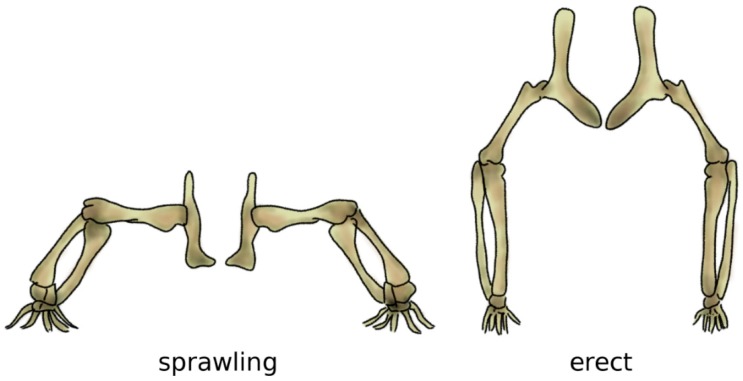
The sprawling (left) and erect (right) postures. In the sprawling posture, the long bones of the limbs are mostly loaded in bending, whereas the load becomes mostly compressive when standing erect. Adapted from Reference [76].

Bipedalism evolved independently several times in vertebrates, resulting in distinct biomechanical strategies which can achieve it in an energy-efficient manner [73]. In archosaurs, i.e., the group which includes crocodilians and dinosaurs, as well as birds, it is believed that bipedalism evolved in parallel with gigantism, since long bones are more effective and strong under the high loading resulting from the body weight if their main axis is aligned with gravity, i.e., vertical [74]. In fact, bone tissue has high mechanical strength in compression but is more susceptible to fractures if loaded in bending, which would result from a horizontal alignment like the one resulting from the sprawling posture common in the ancestors to dinosaurs [75] (Figure 6).

In dinosaurs, forelimbs are relatively underdeveloped with respect to hindlimbs, which retain the major role in standing and locomotion [77]. Even if they employed an erect posture, the spine in the trunk maintained a horizontal alignment; the weight of the trunk was counterbalanced by a massive tail so that the center of mass of the body roughly corresponded with the hip joint [78]. Indeed, in early archosaurs, the spine was not significantly impacted by the adoption of the erect posture. Pseudosuchians, i.e., crocodiles and their ancestors, did not further develop bipedalism and later even reverted to a quadruped sprawling terrestrial gait, in agreement with their semi-aquatic life as ambush predators [78]. On the contrary, the early ornothodirans, the common ancestors of birds, developed a highly flexible neck and a stiff low back (the synsacrum), which are still observable in birds [78]. Trunk alignment in birds is indeed still generally horizontal, and the static equilibrium of the body is facilitated by the lightness of their hollow bones [67,79].

Primate bipedalism, particularly in humans, involves different mechanisms with respect to the erect posture of dinosaurs and birds. Indeed, when a primate is in the erect posture, its spine is mostly vertical; therefore, there is no need for a heavy tail counterbalancing the weight of the trunk [72]. These novel postures required profound adaptations of the spinal anatomy with respect to non-bipedal mammals, which have a spinal anatomy designed to permit quadrupedal locomotion [72].

**Figure 7:**
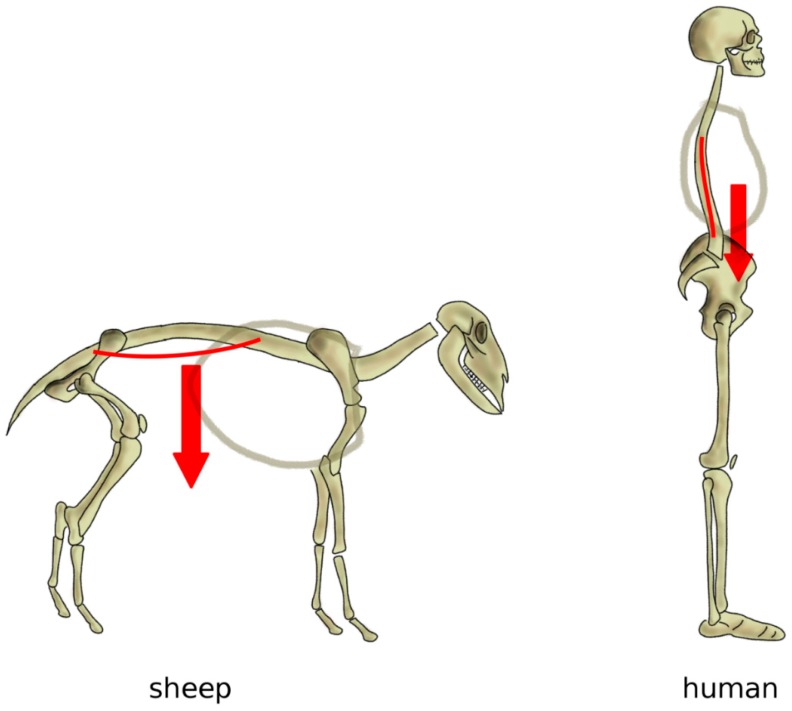
Simplified representation of the action of the body weight in quadrupeds (left) and in bipeds with a vertical spine (right). In quadrupeds such as the sheep, the body weight generates an extension load on the spine resulting in sag, whereas, in a vertical spine, body weight and muscles induce a mostly axial loading in compression.

As mentioned above, in a horizontal posture, the weight of the trunk induces an extension loading on the spine, especially in the lumbar region, whereas a vertical spine is mostly loaded in compression [80] (Figure 7). Indeed, several studies conducted on human cadaver spines, as well as on patients with telemeterized implants, showed that the spinal loading in human standing is well represented by a purely compressive follower load, i.e., a compressive load which follows the curvature of the spine [81,82,83]. Measurements conducted on sheep in vivo and in vitro showed that, although the intradiscal pressures were similar or even higher than those in human spines, compressive forces ranged between 58 and 130 N, versus 400–600 N in humans with comparable body weight [84]; therefore, in quadrupeds, non-compressive loads play a major role.

**Figure 8:**
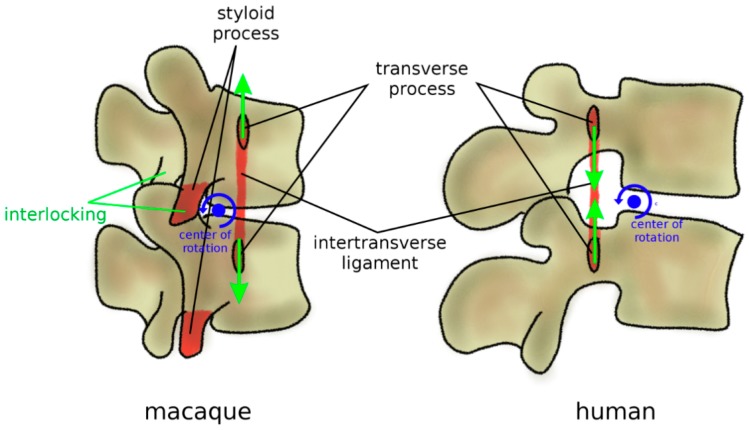
Anatomical protection mechanisms to avoid hyperextension in mammal quadrupeds and non-bipedal primates (macaque, left) in comparison with humans (right). Ventral transverse processes determine tensile stress in the intertransverse ligament in extension, which limits the vertebral rotations. Lumbar vertebrae commonly possess a styloid process which acts as an osseous block to facet sliding in extension. In humans, these protection mechanisms were lost; transverse processes are dorsally located, and the styloid process is absent. Furthermore, facet joints have fewer encompassing shapes and do not strongly interlock in extension.

In addition to zygapophyses, mammal quadrupeds developed three anatomical protection mechanisms to avoid harmful hyperextension, where a large extension motion can result in pinching and excessive strain of the neural structures (Figure 8). Firstly, the transverse processes are most commonly ventrally located [80]; therefore, an extension motion creates a tensile stress in the intertransverse ligament, which limits the vertebral rotations. Secondly, facet joints present curved, encompassing articular surfaces, which tend to strongly interlock if loaded in extension [85]. Thirdly, in most quadrupeds and monkeys, lumbar vertebrae possess a styloid process which acts as an osseous block to facet sliding in extension [80]. In apes and humans, all three anatomical features were lost (Figure 8); transverse processes are dorsally located with respect to the center of rotation in extension, the interlocking of the facet joints in extension does not occur, and the styloid process is absent [80]. Therefore, the spines of humans and apes appear to be unable to sustain a habitual quadrupedal gait and locomotion, and they are, therefore, designed to be subjected to mostly axial loads as those found during brachiation and bipedalism [85].

**Figure 9:**
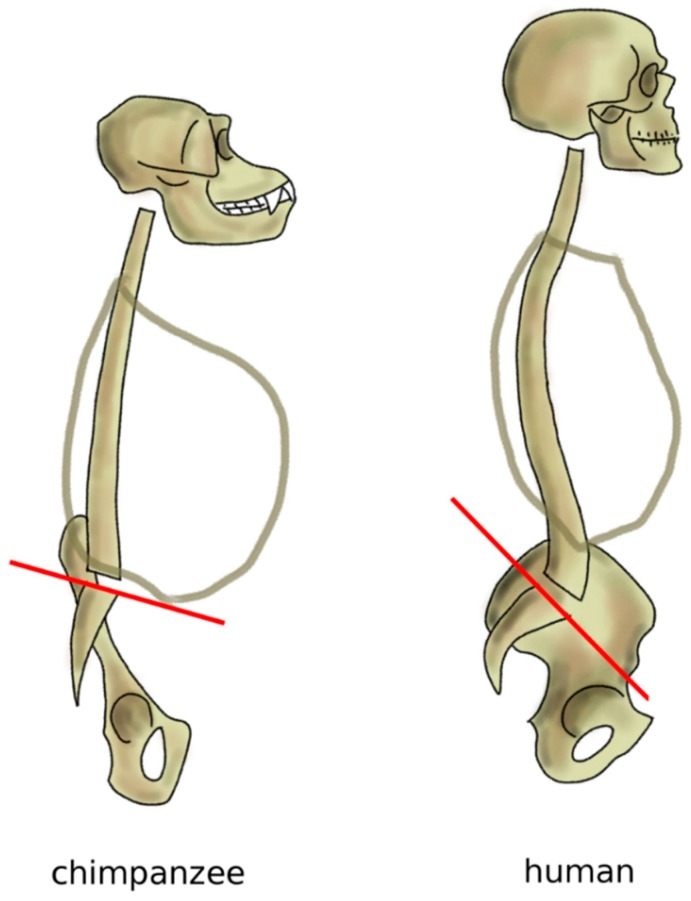
Sagittal profile of the spine in chimpanzees in standing posture and humans. Chimpanzees have an almost horizontal sacral plate, which forces the spine to assume a C-shape. In humans, the higher sacral slope determines the development of an S-shape.

Nevertheless, the spine of great apes such as chimpanzees, bonobos, and mountain gorillas is not fully optimized for the erect posture; indeed, although these primates conduct a mostly terrestrial life, their anatomies are also well adapted to climbing trees and brachiation, which are major activities in their daily lives [86,87]. Apes have an elongated pelvis and a single continuous curvature of the spine, thereby assuming a C-shape [72]. When standing erect, the center of mass of the trunk in chimpanzees is located anteriorly to the hip joints and to the sacrum, resulting in a forwardly imbalanced standing posture, which is partially counterbalanced by appropriate positioning of the forelimbs (Figure 9). This alignment results in high energy expenditure during bipedal gait, which is, therefore, seldom used, and only for short routes [86,88]. Skeletal signs of a habitual erect posture appeared after the split between the other extant apes and humans, 5–11 million years ago [89]. Findings of lumbar vertebrae of australopithecines show a higher degree of wedging (the angle between the superior and inferior endplates of the vertebral body) with respect to extant great apes, compatible with a lumbar lordosis (the angle between the superior endplate of the most cranial lumbar vertebra and the inferior endplate of the most caudal one in the standing posture) of approximately 41° [90,91], fairly similar to that of modern humans (51° on average). Interestingly, findings of *Homo neanderthalensis* revealed smaller lordotic angles (around 29°), which might be related to postures and locomotory strategies distinct from those of modern humans [90,91]; researchers hypothesized that the lower lordosis might be advantageous in walking on sloped terrain, which was likely common in their natural habitat [92]. In parallel to the increase of lumbar lordosis, hominins progressively adapted the shape of the sacrum and pelvis to bipedalism (Figure 9). Whereas the sacral endplate is horizontal in chimpanzees and mountain gorillas in standing, in humans, it has a slope typically between 30° and 60° [93]. It is interesting to note that the pelvic incidence of the human fetus was shown to be comparable to that of the chimpanzees, whereas the distinguishing features of the pelvic shape are developed only after birth [94].

**Figure 10:**
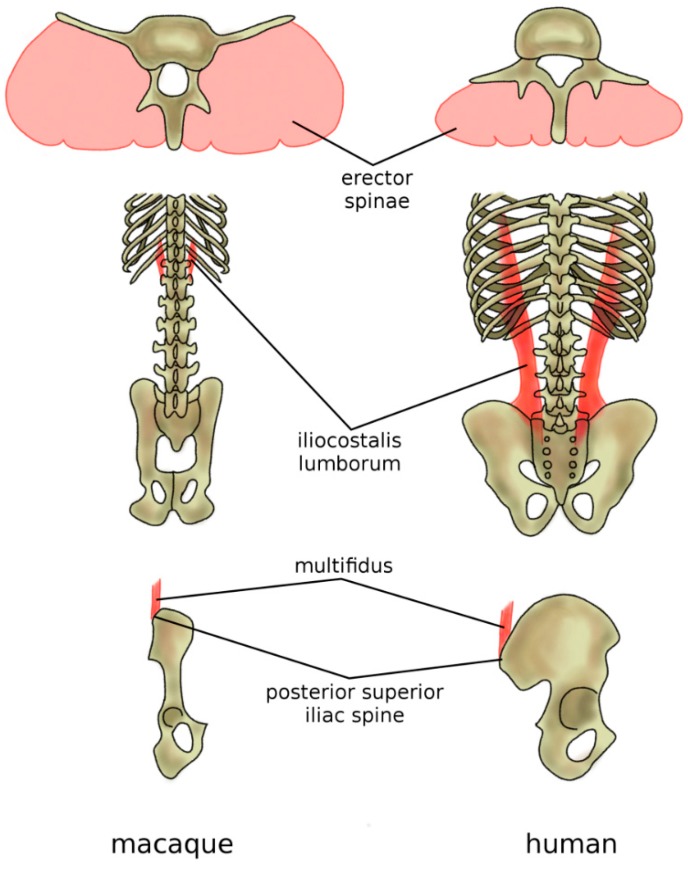
Adaptations of the back musculature of humans (in comparison with macaques) to allow for bipedal stance and gait. In humans and all other hominoids, the erector spina are dorsally located and functions, therefore, as an extensor (top), whereas, in the macaque, it is more ventrally located and has only a minor extensor function. The caudal insertion of the iliocostalis lumborum moved from the lumbar spine to the iliac crest (middle), thus enhancing its lateral flexor function. Specifically, in humans, the posterior superior iliac spine which acts as the iliac insertion of the multifidus is markedly more posterior, thus providing an additional lever arm and room for muscle mass (bottom).

The adoption of the erect posture and bipedal gait induced adaptations in the musculature of the back. In hominoids, the strongest back muscles, namely, the erector spinae (iliocostalis, longissimus, spinalis), together with the multifidus, are dorsally located with respect to the vertebrae and function, therefore, as extensors [95,96] (Figure 10). Their mechanical role is counterbalanced by the rectus abdominis, which is located ventrally with respect to the spine and abdomen and, therefore, has a considerable lever arm when acting as a spine flexor [97]. Indeed, typical human activities such as walking, running, lifting weights, and carrying them in front of the trunk need proper capability in supporting and balancing the weight of the trunk [98], and their back muscles present anatomical peculiarities which improve their effectiveness in such tasks [99]. The powerful extensor function of the longissimus is made possible by the dorsal repositioning of the lumbar transverse processes [80]. The caudal insertion of the iliocostalis lumborum moved from the lumbar spine to the iliac crest, thus enhancing its lateral flexor function which is fundamental in trunk balancing during upright posture and activities [80] (Figure 10). The latter adaptation may have evolved to facilitate brachiation rather than bipedal gait, but resulted also to be very valuable after hominins abandoned arboreal locomotion. Finally, specifically in humans, the posterior superior iliac spine which acts as the iliac insertion of the multifidus is markedly more posterior, thus providing an additional lever arm and room for muscle mass [80,99] (Figure 10).

## 3. Embryology and Development of the Spine

The study of embryos reveals striking analogies with evolution and is indeed used as a powerful tool to gain a better understanding of evolution itself. In their early stages, all vertebrate embryos are structurally very similar; for example, they all have gill slits, even if in terrestrial animals; gills do not further develop and disappear before birth [100]. The next paragraphs provide a brief summary of vertebrate embryology, with a special focus on the development of the spinal column.

The origin of the spine takes place in the early stages of gestation (in humans, during the third and fourth weeks), when the blastula, an agglomerate of cells shaped as a hollow sphere surrounding a fluid-filled cavity, develops into the gastrula, a structure consisting of three differentiated germ layers named the ectoderm, mesoderm, and endoderm [101]. During gastrulation, i.e., the transition between blastula and gastrula, the main directions of the body (in humans and other animals, the cranio-caudal and the dorsal–ventral axes) emerge. The notochord and the somites which later develop into the vertebrae also appear in the gastrulation phase [102].

In most vertebrates, the notochord tends to disappear or to be largely reduced in the adult age, leaving the biomechanical function of supporting the body weight to the vertebral column [103]. As mentioned previously, in some phylogenetically older vertebrates such as jawless fish, the notochord retains its main structural function of body support throughout life [23].

In the embryos of amniotes, the development of the notochord is a complex process and involves the formation of a primitive streak, a band of cells which first appears at the caudal end of the embryo and then develops toward the cranial direction, ending in the primitive knot [100,102,104]. From there, the migration of mesenchymal cells in the direction of the prechordal plate, the site of the future mouth, creates a band of cells named the notochordal process, which then develops into the notochord [100,102].

Lateral to the notochord, the mesodermal cells differentiate, resulting in the formation of 42 to 44 somites aligned along the cranio-caudal axis, which later develops into the axial skeleton and relevant muscles [102,104]. Each somite divides into a sclerotome, which then develops into vertebrae and ribs, dermomyotome (dermis and skeletal muscles), myotome (skeletal muscles), and syndetome (tendons) [100].

**Figure 11:**
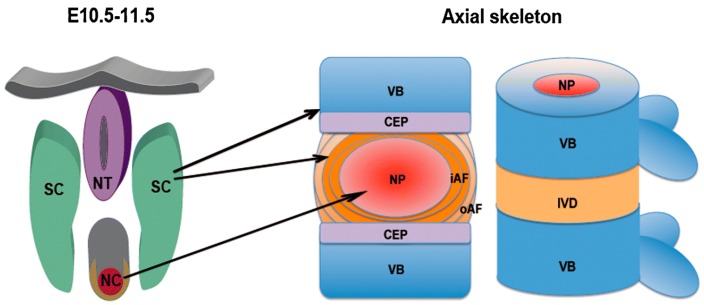
Schematic representation of the development of vertebrae and intervertebral discs from the notochord and the sclerotome. Left: mouse embryo at day 10.5–11.5 of development; right: mature axial skeleton. The nucleus pulposus develops from the notochord, whereas the annulus fibrosus and the other tissues including vertebral body, ligaments, and endplates originate from the sclerotome. NT: neural tube; SC: sclerotome; NC: notochord; VB: vertebral body; IVD: intervertebral disc; NP: nucleus pulposus; iAF: inner annulus fibrosus; oAF: outer annulus fibrosus; CEP: cartilaginous endplate. Reprinted with permission from Reference [106].

In the fourth week of human gestation, the cells in the sclerotome migrate toward the notochord, giving rise to the formation of two regions of packed cells, one cranially and one caudally. The cell-free space between the two regions is then filled by cells migrating from the caudal cell-packed area, which forms the annulus fibrosus, whereas the nucleus pulposus develops from notochordal material [105,106]. The appearance and progress of the intervertebral disc forces the cell-packed regions of the adjacent somites to interact, leading to the development of the centrum, and later of the vertebral body (Figure 11) [100,106].

During the same weeks of the formation of the notochord, the neural tube, which is the progenitor of the central nervous system, develops as well, through a folding and successive closure of the ectodermal tissue. Cells of the sclerotome surround the neural tube, later originating the vertebral arches and the posterior elements which have the main function of protecting the spinal cord and the nerve roots [102].

Between the ninth and the 14th weeks of human gestation, three primary ossification centers appear in each future vertebra, one in the centrum and one for each side in the neural arches [107]; the atlas and the axis have a different number and location of the ossification centers due to their peculiar anatomy [108]. Ossification starts at the cervicothoracic junction, and then progresses to the cervical and thoracolumbar vertebrae; ossification centers at the lumbar neural arches are the last to appear [107,109]. At the time of birth, vertebral bodies and neural arches are still not connected; primary ossification is completed in the first year of extrauterine life. Secondary ossification centers develop at puberty in various locations (at the tips of the spinous process and of the transverse processes, and the annular epiphyses of the vertebral bodies in order to create the endplates) [108]; skeletal maturity is then fully achieved only in the young adult age. In adult human subjects, after ossification, the notochord disappears completely, whereas, in other animals, including mammals, notochordal cells can be found even in elderly individuals [110].

## 4. Discussion and Implications for Biomimetics

This paper presents a brief description of the evolution and development of the spine, emphasizing the strategies which emerged in order to fulfill the functions of the vertebral column. These functions, which involve considerable biomechanical challenges, are the protection of the spinal cord from traumas and excessive mechanical strain, the support of the body weight during locomotion and other tasks, and the flexibility and mobility necessary to perform those tasks in the most efficient way. We discussed how the spine of vertebrates, although being constrained by size requirements and the general outline of the body plan, adapts in order to best fit the specific environment and ecology; for example, we described how the transition to terrestrial life, with the loss of buoyant support in the aquatic environment and the consequent burden of the body weight, determined the appearance of protection mechanisms such as interlocking articulations.

In chordate invertebrates such as cephalochordates, the most primordial spine-like anatomical structure, the notochord, acts as a rigid but flexible rod running along the neural tube [111], preventing excessive strains, as well as attachments for muscles [103]. In the aquatic descendants of the first chordates, vertebrae with a rather simple morphology consisting basically of a centrum, neural arches, and spine, together with the remnants of the notochord, provide sturdier but flexible protection; the notochord provides structural support during embryonic development but does not generally persist throughout life [110]. The transition to the terrestrial environment brought together tremendous biomechanical challenges, first and foremost the effect of gravity load. New locomotion strategies were also needed, resulting in the evolution of limbs, as well as of the shoulder and pelvic girdles [34]. In the spine, the necessity of sustaining the body weight while avoiding excessive body motion determined the appearance of specialized structures such as the zygapophyses and a sophisticated ligamentous complex. Specific ecological needs and behaviors determined further specialized changes, such as the increased stiffness of the synsacrum to facilitate flight in birds [60] and the development of a sagittal spine curvature in bipedal hominins [72].

It is evident that the history of spine development and evolution, as presented in this narrative literature review, provides plenty of cues for bionic research. An example of design taking inspiration from the spinal anatomy was recently presented by a research group from Columbia University, who described a flexible lithium battery aimed at powering wearable electronics [112]. Although the authors claimed a resemblance with a human spine, the actual design featuring a flexible core around which stiffer electrodes are wound shares more similarities with the spine of phylogenetically older vertebrates in which a continuous notochord is regularly surrounded by centra. Such design determines a flexibility independent of the direction of loading; in case an anisotropic response is desired, features similar to zygapophyses may be considered.

Articulated spines based on bio-inspired designs are being used in robotics, with the aim of improving stability and maneuverability during locomotion, both in quadrupedal [113] and bipedal designs [114]. Current and foreseen applications of such robots include the biomechanical study of locomotion and its neural control [115], providing a physical framework for the implementation of artificial intelligence solutions [114], military and search-and-rescue missions, space exploration, and outdoor industrial inspection [116]. Recently, bio-inspired robots aimed at the detailed study of animal locomotion [117], including that of extinct species [118], were presented and are currently a subject of intense research. It should be noted that the latter robots feature an active control of the spinal motion by means of electric servomotors which closely replicate the three-dimensional joint movement during locomotion; while passive structures such as intervertebral discs, ligaments, and articular joints are yet to be implemented. there is ample space for further developments.

In a recent paper, the human intervertebral disc was used as a basis for a bio-mimetic construct aimed at the replacement or repair of the intervertebral disc itself, in patients needing surgery for degenerative disorders [119]. The authors developed a biocomposite laminate including long collagen fibers with an orientation mimicking that in the human lumbar disc, embedded in alginate hydrogel. The novel construct was subjected to mechanical testing and revealed properties similar to that of the native tissue in bending, but resulted excessively flexible in torsion, thus indicating the need for further research prior to clinical testing [119]. A simpler, fully mechanical artificial intervertebral disc replacement with a bio-inspired design is currently commercially available for use in both the lumbar and cervical spine [120,121]. This prosthesis consists of a viscoelastic polymeric core simulating the native nucleus pulposus, surrounded by a fiber jacket and a polymeric sheath, mimicking the anatomy and function of the annulus fibrosus. Fixation to the adjacent vertebral bodies is ensured by titanium keeled endplates. Biomechanical testing confirmed a flexibility similar to that of the native intervertebral disc [122].

It is interesting to note that, although thousands of previous papers investigated in detail the biomechanics of the human spine, and although animals were frequently used as a model for the investigation of human pathologies and treatments, relatively little research was conducted on the spine biomechanics of most non-human vertebrates for the purpose of understanding it per se. For example, the flexibility of the spine of the sheep, pig, and calf was extensively studied only due to the fact that spine specimens explanted from these animals are commonly employed for in vitro testing of spinal implants as a surrogate of human specimens [123,124,125]. With the notable exception of some studies in the field of veterinary surgery (e.g., References [126,127]), as well as pure basic research papers (e.g., References [59,78,128]), spine biomechanics of non-human vertebrates appears to be significantly less intensively investigated than in humans. Nevertheless, even animals which are perceived as “primitive” due to their phylogenetic distance from humans have anatomies which are highly optimized for their ecological niche and, thus, have enormous biomimetic potential.

In summary, we firmly believe that the evolution of vertebrates describes countless examples of solutions which effectively address challenging biomechanical problems, enabling them to act as valuable starting points for technicians and developers working in bionic engineering. In particular, the history of the spine constitutes a vast source of opportunities for possible ideas and innovations, especially in the field of structural engineering.
